# Metagenomics-Based Approach to Source-Attribution of Antimicrobial Resistance Determinants – Identification of Reservoir Resistome Signatures

**DOI:** 10.3389/fmicb.2020.601407

**Published:** 2021-01-15

**Authors:** Ana Sofia Ribeiro Duarte, Timo Röder, Liese Van Gompel, Thomas Nordahl Petersen, Rasmus Borup Hansen, Inge Marianne Hansen, Alex Bossers, Frank M. Aarestrup, Jaap A. Wagenaar, Tine Hald

**Affiliations:** ^1^Division of Genomic Epidemiology, National Food Institute, Technical University of Denmark, Kgs Lyngby, Denmark; ^2^Institute for Risk Assessment Sciences, Faculty of Veterinary Medicine, Utrecht University, Utrecht, Netherlands; ^3^Intomics A/S, Lyngby, Denmark; ^4^Wageningen Bioveterinary Research, Lelystad, Netherlands; ^5^Department of Infectious Diseases and Immunology, Faculty of Veterinary Medicine, Utrecht University, Utrecht, Netherlands

**Keywords:** metagenomics, source-attribution, antimicrobial resistance, resistome, random forests, machine learning

## Abstract

Metagenomics can unveil the genetic content of the total microbiota in different environments, such as food products and the guts of humans and livestock. It is therefore considered of great potential to investigate the transmission of foodborne hazards as part of source-attribution studies. Source-attribution of antimicrobial resistance (AMR) has traditionally relied on pathogen isolation, while metagenomics allows investigating the full span of AMR determinants. In this study, we hypothesized that the relative abundance of fecal resistome components can be associated with specific reservoirs, and that resistomes can be used for AMR source-attribution. We used shotgun-sequences from fecal samples of pigs, broilers, turkeys- and veal calves collected across Europe, and fecal samples from humans occupationally exposed to livestock in one country (pig slaughterhouse workers, pig and broiler farmers). We applied both hierarchical and flat forms of the supervised classification ensemble algorithm Random Forests to classify resistomes into corresponding reservoir classes. We identified country-specific and -independent AMR determinants, and assessed the impact of country-specific determinants when attributing AMR resistance in humans. Additionally, we performed a similarity percentage analysis with the full spectrum of AMR determinants to identify resistome signatures for the different reservoirs. We showed that the number of AMR determinants necessary to attribute a resistome into the correct reservoir increases with a larger reservoir heterogeneity, and that the impact of country-specific resistome signatures on prediction varies between countries. We predicted a higher occupational exposure to AMR determinants among workers exposed to pigs than among those exposed to broilers. Additionally, results suggested that AMR exposure on pig farms was higher than in pig slaughterhouses. Human resistomes were more similar to pig and veal calves’ resistomes than to those of broilers and turkeys, and the majority of these resistome dissimilarities can be explained by a small set of AMR determinants. We identified resistome signatures for each individual reservoir, which include AMR determinants significantly associated with on-farm antimicrobial use. We attributed human resistomes to different livestock reservoirs using Random Forests, which allowed identifying pigs as a potential source of AMR in humans. This study thus demonstrates that it is possible to apply metagenomics in AMR source-attribution.

## Introduction

Source-attribution estimates the proportion of human cases of a foodborne disease attributable to different reservoirs and/or vehicles of transmission, i.e., different sources (Pires et al., 2018). Microbial subtyping studies of source-attribution have successively contributed in many countries to distribute the burden of specific foodborne diseases by animal reservoirs (Hald et al., 2004; Mughini-Gras and van Pelt, 2014; Mughini-Gras et al., 2019; Pires et al., 2014; de Knegt et al., 2016; Thépault et al., 2018; Zhang et al., 2019). Attribution estimates can be obtained using different data inputs and distinct modeling approaches (Mughini-Gras et al., 2018), but traditionally many single-pathogen targeted studies have relied on frequency-matching models and phenotypic data (Hald et al., 2004; Mughini-Gras and van Pelt, 2014; de Knegt et al., 2016). However, with the greater availability of genotyping information of foodborne pathogens isolated from animals, food, the environment and clinical cases, population genetic models have become an increasingly popular choice (Thépault et al., 2018), and several model developments have been seen in order to accommodate whole genome sequencing (WGS) data (Cheng et al., 2013; Raj et al., 2014; Lees et al., 2019; Tonkin-Hill et al., 2019). At the same time, machine learning has been exploited as an alternative approach to perform source-attribution with WGS (Zhang et al., 2019; Munck et al., 2020). For example, a machine learning decision tree algorithm has been recently proposed for the attribution of *Salmonella* Typhimurium infections in humans using core-genome multilocus sequencing (Munck et al., 2020), with results comparable to those previously obtained when combining Multiple Locus Variable-number Tandem Repeat Analysis (MLVA) data with the Hald frequency-matching model (de Knegt et al., 2016).

The transmission of foodborne pathogens to humans implies some degree of concomitant transmission of antimicrobial resistant (AMR) bacteria and AMR determinants. The magnitude of such transmission has, however, been only limitedly estimated to date, usually targeting a single combination of pathogen-resistance type, e.g., ESBL-AmpC-producing *E. coli* (Evers et al., 2017; Mughini-Gras et al., 2019), or by attributing AMR infections to sources, proportionally to attribution of pathogen subtypes and the prevalence of phenotypic resistance in each subtype (Hald et al., 2007). Traditionally, source attribution has been strongly based on modeling transmission from animals to humans, but recently, human-to-human transmission has been successfully incorporated in a frequency-matching model (Mughini-Gras et al., 2019), which showed a high relative contribution of that transmission route to human infections with ESBL-AmpC- producing *E. coli.*

Due to the genetic nature of antimicrobial resistance, and the possibility of horizontal transfer of resistance genes between bacteria of different species, we propose here to assess transmission of AMR by considering the abundance of genetic determinants of resistance derived from metagenomic sequencing (i.e., the resistome). Metagenomics has indeed been previously demonstrated to be a valuable asset in trace-back studies to determine the origin of AMR contamination in water bodies (Baral et al., 2018; Gupta et al., 2019). We used the fecal resistomes from broilers, pigs, turkeys and veal calves sampled close to slaughter among nine European countries, in order to identify reservoir-specific resistome signatures and use them to predict the sources of the fecal resistome of humans with occupational exposure to livestock. Human samples included subjects working on pig farms, pig slaughterhouses or broiler farms in one of the participating countries (Van Gompel et al., 2020). We compared the predictions of models with and without the inclusion of “humans” as a source of AMR determinants, and the predictions of country-independent and country-specific models.

This study allowed to predict the relative attribution to different animal reservoirs of AMR determinants present in human resistomes, and thus sets the scene for future source-attribution studies of AMR transmission using metagenomics.

## Materials and Methods

All samples used in this study were collected during cross-sectional studies under the scope of the European project EFFORT^1^. Sampling protocol, number of samples collected, DNA extraction, sequencing method and diversity analysis of the resistomes have been described in detail elsewhere (Munk et al., 2018; Van Gompel et al., 2020). Sampling occurred among nine participating countries, which were anonymized with letters A-I, as agreed by the project consortium.

### Sample Collection

**FIGURE 1:**
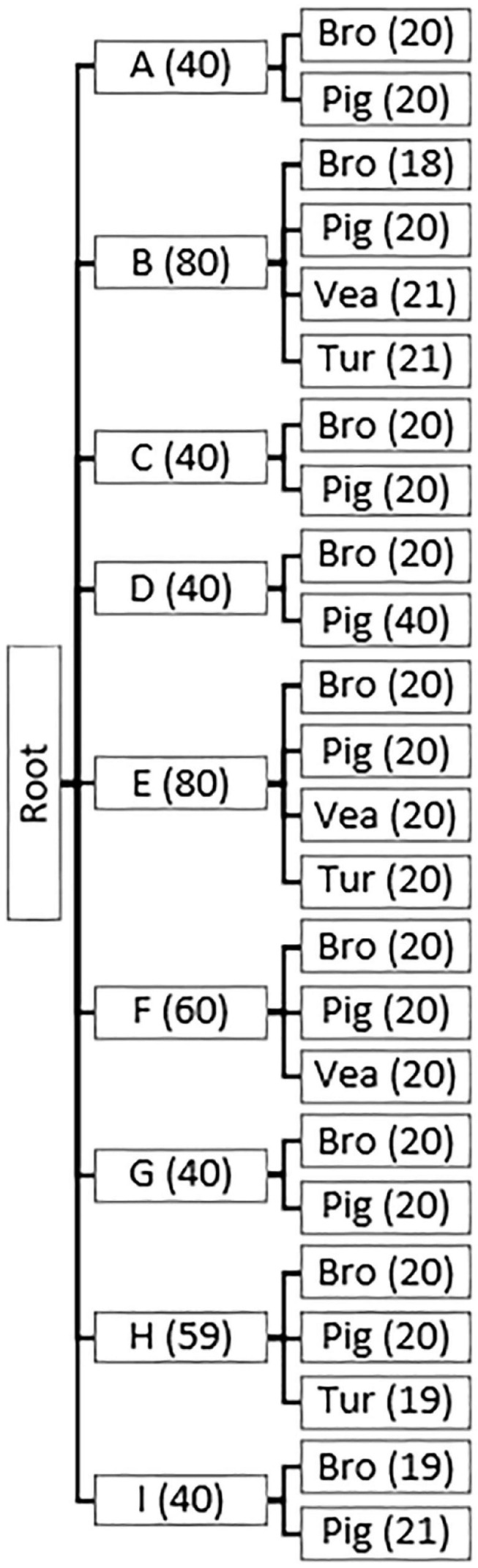
Parent and child nodes in Hierarchical Random Forests models (HRF1, HRF2). Hierarchical structure of the models HRF1 and HRF2, with parent nodes (countries) and child nodes (reservoirs within a country). Number of observations per country **(A-I)** and per reservoir (Bro = broiler, Pig = pig, Vea = veal calves, Tur = turkey) is indicated between brackets. One sample corresponds to a pool of 25 individual samples from a single farm.

Shortly, a total of 25 pen-floor fresh fecal samples were collected from and pooled in a single pool for each sampled pig-, broiler-, turkey- and veal calves- farm. Pig- and broiler-farms were sampled in nine European countries (A-I), and turkey and veal farms in three among those nine countries (Figure 1). Individual fecal samples from humans with occupational exposure to food-producing animals were collected in country F, with each sample representing an individual subject. The characteristics of the sampled broiler farm workers (*n* = 24), pig farm workers (*n* = 54) and pig slaughterhouse workers (*n* = 70) have been described elsewhere (Van Gompel et al., 2020). Here we use the single term ‘workers,’ however, samples collected from humans on pig and broiler farms included both farmers, their family members and employees.

### Shotgun Sequencing

The procedures for DNA extraction and shotgun sequencing were described in detail elsewhere for the animal samples (Munk et al., 2018) and for the human samples (Van Gompel et al., 2020). Shortly, samples from pigs and broilers were mostly sequenced on the Illumina HiSeq3000 platform (provider Oklahoma Medical Research Foundation), samples from turkeys and veal calves were sequenced on the Illumina NovaSeq 6000 platform (provider Admera Health), and samples from humans were sequenced on the Illumina HiSeq4000 platform (provider GenomeScan), all using 2 × 150-bp paired-end sequencing per flow cell. Library preparation for broiler and turkey samples involved PCR amplification, whereas for pig, calf and human samples it was amplification-free.

### Read Mapping

The bioinformatic analysis of the metagenomic raw reads was similar to the one described by Munk et al. (2018), with the use of a more recent version of the ResFinder database containing 3026 reference sequences of AMR genes (accessed on 21st September 2018). Shortly, DNA sequences from each sample were analyzed with MGmapper (Petersen et al., 2017). In order to avoid PCR copies in the poultry and turkey data, identical read pairs were removed using Picard v2.8.3^2^. Adaptor sequences and low-quality nucleotides were also removed using BBduk2^3^. The resulting trimmed read pairs were aligned to the ResFinder database^4^ described by Zankari et al. (2012) using the Burrows-Wheeler Aligner (Li and Durbin, 2009). Properly paired reads, with at least a 50-bp alignment in each read, were accepted. The resulting mapped reads represented the acquired resistome in each sample.

### Normalization of Read Counts

ResFinder-mapped counts were normalized to the length of each reference sequence as well as to the number of reads in each sample, thus converting counts into values of Fragments Per Kilobase of reference and Million reads (FPKM) for each ResFinder reference sequence. Genes with many alleles in ResFinder result in unspecific mapping and randomly assigned read pairs. To avoid sensitivity loss and wrong assignments, we kept ambiguous hits, but aggregated their abundances to higher levels, corresponding to 90% gene identity clusters (herein referred to as AMR determinants). To determine these clusters, we used CD-HIT-EST (v4.6.6) (Li and Godzik, 2006; Fu et al., 2012) at a 90% identity level and otherwise default settings. The resulting read count matrix contained 389 individual AMR determinants. An overview of the gene-level composition of each AMR determinant is provided in Supplementary Table 1.

### Random Forests

We applied the supervised classification ensemble algorithm Random Forests (RF) (Ho, 1995; Breiman, 2001) in order to classify resistomes into corresponding reservoir classes, based on their relative composition of AMR determinants. According to Breiman (2001), Random Forests is “a classifier consisting of a collection of tree-structured classifiers (…) (where) each tree casts a unit vote for the most popular class” for each observation and “after a large number of trees is generated, they vote for the most popular class” overall, also called the ‘crisp’ class. The splits within each tree are determined based on a random selection of features, which is believed to improve model accuracy (Breiman, 2001). In its flat form, Random Forests compares one against all classes (Gavish et al., 2018) for each observation, in this study, one against all reservoirs. We applied in a first step hierarchical Random Forests (HRF), which considers a hierarchical data structure and uses flat RF algorithms at each internal hierarchy node. In this step, observations were classified both in terms of the reservoir and of the country of origin. In HRF, each internal node classifier is trained exclusively on observations of descendants of that particular node (Gavish et al., 2018), thus here, classification of an observation in terms of the reservoir was trained separately within each country node.

All analyses were performed in R v. 3.6.0 (R Core Team, 2019): Hierarchical Random Forests with the package *HieRanFor* v.1.0^5^ and Random Forests with the package *caret* v.6.0–84 (Kuhn et al., 2019).

**FIGURE 2:**
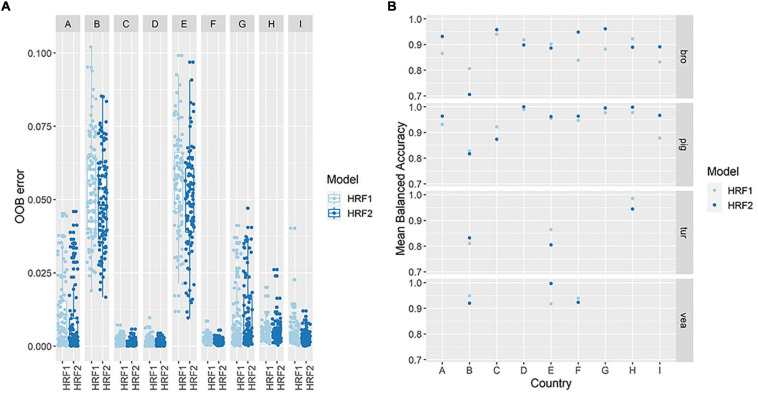
Performance of Hierarchical Random Forests with full feature space (HRF1) and with reduced dimensionality (HRF2). *Model performance measures for HRF1 and HRF2 include* out of bag (OOB) errors at parent nodes (individual country) **(A)** and mean balanced accuracy at terminal nodes (reservoir) **(B)**. The figure shows the distribution of both measures obtained with 100 iterations of HRF1 (full feature space, i.e., 389 AMR determinants) and HRF2 (reduced feature space, i.e., 119 selected AMR determinants).

We fit a total of five models in the study, termed HRF1, HRF2, RF1, RF2 and RF3. In each model, we set the parameter *mtry* (number of features available for splitting at each tree node) as the square root of the number of predictor features (AMR determinants), and we initially split the data into a training and a testing subset, corresponding to random 70% and 30% of the total number of observations, respectively. Model performance was assessed on the basis of balanced accuracy (Kuhn et al., 2019) and Kappa value (Cohen, 1960) for all models, and in terms of out of bag error (OOB) (Gavish et al., 2018) at the HRF parent nodes, (Figure 2 and Supplementary Table 2). The predictive performance of each model was further assessed both with the training set (self-attribution) and with the testing set. Predictions for each observation consisted on i) the proportion of votes given to different reservoir classes, here interpreted as the relative probabilities of a sample being attributed to different reservoirs, and ii) a crisp class. The importance of each AMR determinant for class attribution was assessed based on the mean decrease in accuracy (MDA), i.e., the decrease in the model’s accuracy resulting from removing that individual feature.

Further details of each model are given in sections 2.5.1 to 2.5.5.

#### HRF1 – Identification of Country-Independent, Reservoir-Discriminant Resistome Signatures

The first step of the analysis, the hierarchical Random Forests HRF1, considered the full animal resistome dataset, consisting of 479 observations and 389 AMR determinants, with observations distributed among 9 countries (A – I) and 4 reservoir classes (pig (181), broiler (177), veal calves (61), turkey (60)). Two hierarchy levels were defined, L1 = ‘country’ and L2 = ‘reservoir’ and the data was considered balanced at the level of the reservoir class within each country (the terminal nodes), with approximately 20 farms sampled per reservoir, per country (Figure 1). The model was run with 10× Monte Carlo (MC) random sampling validation and predictions were obtained for the training set and the testing set in each iteration. Random training and testing subsets were drawn in each MC iteration. For each node in the hierarchy, the list of AMR determinants and their MDA for classification of the node’s descendants was extracted. Each list was subsequently filtered to those AMR determinants which contributed positively (MDA > 0) to the classification.

In order to define country-independent resistome signatures, we investigated the overlap of the country-specific subsets of AMR determinants between different groups of countries: i) 5 countries with pig and broiler observations only, ii) 4 countries with observations of pig, broiler, turkey and/or veal calves and iii) 2 countries with observations of all four reservoirs. The overlaps were determined and visualized in Venn diagrams with the R package *VennDiagram* v.1.6.20 (Chen, 2018; Supplementary Figure 1), and were the basis to define the subsets of country-independent and country-specific resistome markers, used in subsequent steps of the analysis.

The aim with HRF1 was to identify country-specific and -independent AMR determinants with a positive impact on the classification of animal resistomes into corresponding reservoirs. The results of HRF1 informed the dimensionality reduction (reduction of the number of features in the model) performed in subsequent steps (HRF2, RF1, RF2, RF3).

#### HRF2 – Model Performance With Country-Independent AMR Determinants

We selected a set of 119 AMR determinants with MDA > 0 determined in HRF1 for the two countries where all four reservoirs were sampled. This country-independent subset of AMR determinants defined the feature space in HRF2. These AMR determinants allow distinguishing between the resistomes of animal reservoirs across all included countries. HRF2 was trained and assessed equally to HRF1. The aim of HRF2 was to assess the change in model performance after a targeted dimensionality reduction step. In order to compare the performance of HRF1 and HRF2, we investigated the percentage of concordance between the crisp class and the true class, the list of AMR determinants with positive MDA and the OOB errors at each country node, and the balanced accuracy at each terminal node (country-reservoir).

#### RF1 – Source-Attribution of Human Resistomes With an Animal Resistome Model

The same data used in HRF2 was randomly upsampled in order to achieve a balanced number of observations between reservoir classes. Upsampling was performed with the function *upSample* from the R package *caret* v.6.0–84 (Kuhn et al., 2019), which samples with replacement until class distributions are equal. A flat RF model was then trained with no hierarchical levels considered. First, the data was split into a training and a testing set and then the algorithm was trained with 10 × 10-fold cross validation (i.e., 10-fold cross validation repeated 10 times) on the training set. Predictions were obtained for the initial training set and testing set, and additionally for all 149 observations of human resistomes separately. This model allowed attributing human resistomes to different animal reservoirs in a probabilistic manner, by considering ‘pig,’ ‘broiler,’ ‘turkey,’ and ‘veal’ as the only possible sources of AMR determinants.

#### RF2 – Source-Attribution of Human Resistomes Including Human as a Source

Following RF1, a similar RF model was developed by adding the human samples to the initial dataset, thus allowing classification votes to the class ‘human,’ additionally to the classes ‘pig,’ ‘broiler,’ ‘turkey,’ and ‘veal.’ Before random splitting into a test and a training set, the data was upsampled as described for RF1 in order to achieve data balance between classes. Then, the RF2 algorithm was also fit with 10 × 10-fold cross validation to the training set, and predictions were obtained for the initial training and testing sets, and exclusively for the human observations. RF2 allowed the attribution of human resistomes to a source alternative to the four food-producing animals considered in the previous models. ‘Human’ as a source represents an origin of AMR determinants that cannot be attributed to one of the four animal reservoirs here considered.

#### RF3 – A Country-Specific Source-Attribution Model

A third flat RF model was fit to all observations (animal and human) originating from country F, where the three groups of humans were sampled. RF3 considered thus an initial dataset with a total of 230 observations, distributed among the reservoirs ‘pig’ (20), ‘broiler’ (20), ‘veal’ (20), ‘turkey’ (21) and ‘human’ (149). Since turkey farms have not been sampled in country F, we assumed that consumers in country F are exposed to turkeys with resistomes similar to those that were sampled in country B, which represents the major turkey meat provider to country F^6^^,^^7^. This initial dataset was upsampled, as described for RF1 and RF2 and then split into a training and a testing set. The feature space included the subset of 109 AMR determinants identified in HRF1 with MDA > 0 for reservoir classification within country F (see Supplementary Table 4). The algorithm was fit to the training set with 10 × 10-fold cross validation and predictions were obtained for the initial training and testing sets, and exclusively for the human observations. The model RF3 aimed at identifying the impact of applying country-specific reservoir resistome signatures for the source attribution of human resistomes.

### Reservoir-Indicator AMR Determinants

We performed a similarity percentage analysis (SIMPER) (Clarke, 1993) in order to discriminate reservoir-indicator AMR determinants. We used pairwise comparisons of reservoirs’ resistomes and determined the average contribution of each AMR determinant to the Bray-Curtis dissimilarity between every two reservoirs. This analysis was performed with the full spectrum of AMR determinants using the function *simper* from the R package *vegan* v.2.5–5 (Oksanen et al., 2019). The AMR determinants contributing to the reservoir pairwise dissimilarity were selected and annotated by AMR class and representative AMR gene (see Supplementary Table 4). In each pairwise comparison, the top 10 contributors were selected and the reservoir in which their relative abundance in the resistome was highest was identified. The resistomes were further hierarchically clustered based on Euclidean distances and the Ward’s minimum variance method (Ward, 1963), applied to the Hellinger-transformed relative abundances of those top contributing AMR determinants, using the function *decostand* from the R package *vegan* v.2.5–5 (Oksanen et al., 2019) and the R package *pheatmap* v. 1.0.12 (Kolde, 2019; Supplementary Figure 4). An identical analysis was performed to identify AMR determinants contributing to the resistome dissimilarity between each human group and the animal reservoir to which it was occupationally exposed, i.e., broiler or pig.

## Results

### Selection of Country-Independent Resistome Signatures

With the first hierarchical Random Forests model, HRF1, we identified a subset of AMR determinants that allows an accurate classification of a resistome into its animal reservoir, independently of the country of origin. This was achieved by identifying those AMR determinants with a positive mean decrease in accuracy concurrently in i) two countries where all four reservoirs were sampled (119 determinants), ii) four countries where more than two reservoirs were sampled (77 determinants), iii) five countries were only broilers and pigs were sampled (35 determinants) (Supplementary Figure 1 and Supplementary Table 3). We observed that the number of predictor features necessary to classify a resistome across countries increased with a larger heterogeneity of reservoirs among the samples, however, all features important to differentiate between pig and broiler resistomes only were also important classifiers in the presence of additional reservoirs. The number of country-independent, reservoir-discriminant resistome features varied slightly between cross-validation runs, however, features with top MDA remained unchanged. Therefore, we chose the output from a random iteration of HRF1, to proceed to the training of HRF2.

The performance of the HRF2 model with dimensionally reduced data (119 AMR determinants) was compared to the performance of HRF1 with 389 features. The overall model accuracy was lower in HRF2 (mean accuracy = 0.826) compared to HRF1 (mean accuracy = 0.833), however, the distribution of the OOB errors in HRF2 were comparable to those of HRF1 for the classification of reservoirs within countries, for most countries (Figure 2A). Countries G and H presented higher mean/median OOB errors in HRF2, while countries A, B and E presented lower mean/median OOB errors with the reduced model. The balanced accuracy in the classification of each reservoir species within individual countries was also assessed. The mean accuracy in classification increased in 5/9 countries for broiler samples (A, C, F, G, I), 7/9 countries for pig samples (A, D, E, F, G, H, I), 1/3 countries for veal calves (B) and turkey samples (E) (Figure 2B).

Table 1 shows that the concordance between true and predicted reservoir class with HRF2 increased for classification of countries A (8%), C (2.9%), E (7.8%), H (8%) and I (14.9%), and decreased between 5.9 and 23.1% for the classification of remaining countries. The concordance in the classification of reservoir across countries increased for broiler (0.8%) and turkey (20.3%) and decreased for pig (−3.3%) and veal calves (−11.2%).

**TABLE 1:** Concordance between true class and predicted crisp class for training data set (self-attribution) and feature importance, with models HRF1 and HRF2.

Concordance in self-attribution^*a*^		HRF1	HRF2 (HRF2- HRF1)
Percentage concordance for country class:	A	83%	91% (+ 8%)
	B	71%	64% (−7%)
	C	85%	88% (+ 3%)
	D	85%	79% (−6%)
	E	79%	87% (+ 8%)
	F	83%	75% (−8%)
	G	97%	74% (−23%)
	H	83%	91% (+ 8%)
	I	69%	84% (+ 15%)
Percentage concordance for reservoir class:	Pig	88%	84% (−3%)
	Broiler	75%	76% (+ 1%)
	Turkey	59%	79% (+ 20%)
	Veal calves	95%	84% (−11%)
Feature importance^b^		HRF1 (% of total features in the model)	HRF2 (% of total features in the model)

Number of AMR determinants with mean decrease in accuracy > 0 for a set of:	1 country	29 (7%)	5 (4%)
	2 countries	20 (5%)	6 (5%)
	3 countries	12 (3%)	13 (11%)
	4 countries	25 (6%)	15 (13%)
	5 countries	23 (6%)	21 (18%)
	6 countries	16 (4%)	10 (8%)
	7 countries	21 (5%)	12 (10%)
	8 countries	13 (3%)	10 (8%)
	9 countries	32 (8%)	26 (22%)

*Results represent a single random model iteration of HRF1 and HRF2, and predictions for each model’s respective training dataset (i.e., self-attribution performance). HRF1 was trained on the full set of AMR determinants (389), and HRF2 was trained on a dimensionally reduced dataset (119 features). ^*a*^Concordance in self-attribution’ represents the percentage of crisp class predictions of each model (HRF1 and HRF2) in agreement with the true class of an observation, in prediction of country of origin, and in prediction of reservoir. ^*b*^Feature importance’ represents the number (and percentage) of individual AMR determinants in each model that contributed positively (MDA > 0) for classification among 1, 2, 3, 4, 5, 6, 7, 8, or 9 countries.*

Overall, HRF2 represented neither an overall improvement nor an overall decrease in performance compared to HRF1. Nonetheless, HRF2 resulted in a clear decrease in the proportion of AMR determinants important for country-specific classification and an increase in the proportion of those important for classification in a larger number of countries (Table 1). This result indicates that the subset of AMR determinants selected in HRF1 and used as predictors in HRF2 contains a larger relative proportion of country-independent resistome signatures.

### Source-Attribution of Human Resistomes

**FIGURE 3:**
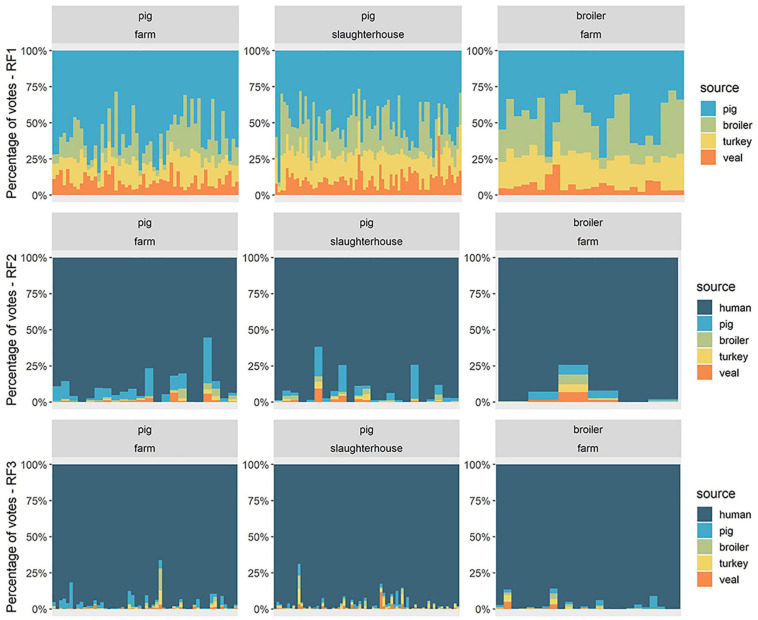
Source attribution of 149 human resistomes with flat Random Forests models. For each human fecal resistome (horizontal axis), the attribution results are presented as the percentage of votes given to each of the reservoir sources (vertical axis). Each horizontal facet represents the predictions of one Random Forests model (RF1, RF2, or RF3). RF1 was trained on resistomes from pig, broiler, turkey and veal calves from all countries; RF2 was trained on resistomes from pig, broiler, turkey, veal calves from all countries and human resistomes from country F. RF3 was trained on resistomes from pig, broiler, veal calves and human sampled in country F and from turkey sampled in country B and considered representative for country F. “Human” as an attributed source represents any other source besides one of the four livestock species included in the model. Each vertical facet represents the occupational exposure setting of the human samples collected in country F – workers on pig farms, pig slaughterhouses or poultry farms.

The models RF1, RF2 and RF3 were used to probabilistically attribute human resistomes to reservoirs, under different hypotheses (see Supplementary Figure 2). RF1 was trained exclusively on animal resistome data, hence human resistomes could only be attributed to the four animal reservoirs. RF2 was trained including both human and animal data, thus human resistomes could additionally be attributed to a ‘human’ source. Both RF1 and RF2 were trained on the set of AMR determinants used previously in HRF2 (i.e., a set of country-independent predictors), and therefore included data from all countries. In terms of crisp class determination, these two models achieved similar accuracy during training (mean accuracy values of 0.996 and 0.995, for RF1 and RF2, respectively (Supplementary Table 2). However, by not including an attribution scenario besides the four livestock sources (i.e., such as the “human” class in RF2), the predictions of RF1 for the source of human resistomes are unrealistic, with the totality of attribution votes distributed exclusively among the sources ‘pig,’ ‘broiler,’ ‘turkey,’ and ‘veal’ (Figure 3). Nonetheless, the attribution results of RF1 indicate a consistently higher proportion of votes attributed to the source ‘pig’ for every human resistome, which suggests a relatively higher similarity between human and pig resistomes, than with the remaining three reservoirs. The predictions with RF2 further showed that every human resistome was in fact predominantly attributed to the source ‘human,’ once the model included that source. The second most attributed source with RF2 was ‘pig,’ in accordance with the attribution pattern seen with model RF1.

**FIGURE 4:**
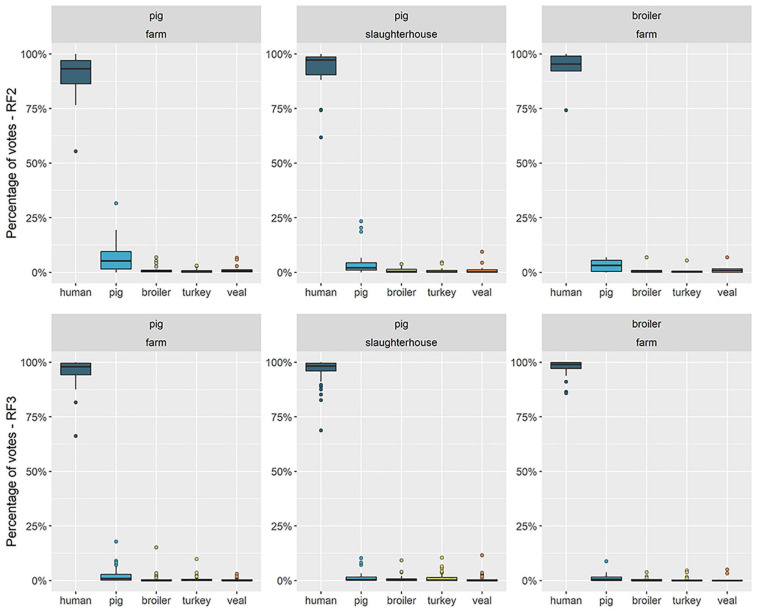
Distribution of percentage of votes attributed to each reservoir in predictions for human resistomes with a country-independent (RF2) and a country-specific (RF3) Random Forests. The boxplots represent the distribution of the percentage of votes (vertical axis) attributed to each reservoir (horizontal axis) among the total of human resistomes. Each horizontal facet represents the predictions of one Random Forests model (RF2 or RF3). RF2 was trained on resistomes from pig, broiler, turkey, veal calves sampled among nine countries and from human sampled in country F; RF3 was trained on resistomes from pig, broiler, veal calves and human sampled in country F and from turkey sampled in country B and considered representative for country F. “Human” as an attributed source represents any other source besides one of the four livestock species included in the model. Each vertical facet represents the occupational exposure setting of the human samples collected in country F – workers of pig farms, pig slaughterhouses or poultry farms.

RF3 was a country-specific model, trained on animal and human samples collected in country F (in the case of turkey, we assumed the resistome of turkeys to be comparable to that observed in country B). The predictor AMR determinants were those identified as specific to country F in model HRF1 (see Supplementary Table 3). RF3 had a higher accuracy during training (0.998) compared to RF2 (0.994) (Supplementary Table 2) and a higher proportion of votes attributed to the true class, in prediction with both testing and training sets (Supplementary Figures 2E,F). The distributions of source votes obtained with RF2 and RF3 among each of the three groups of humans were compared in Figure 4. Among broiler-farm workers, RF3 showed an increase in the proportion of votes for the class ‘human’ at the expense of a decrease in the attribution to the class ‘pig.’ RF3 thus showed that humans exposed to direct contact with pigs had a higher proportion of attribution to ‘pig’ compared to humans exposed to direct contact with broilers. Both RF2 and RF3 showed a higher attribution to ‘pig’ for pig farm workers, compared to pig slaughterhouse workers.

### Analysis of Reservoir-Indicator Resistome Signatures

We assessed the pairwise dissimilarity between reservoirs across all countries and observed that among the four food-producing animals, the highest resistome dissimilarities were between broiler-veal calves and broiler-pig (72.4 and 72.2%, respectively). Opposingly, the two reservoirs with most similar resistome compositions were pig and veal calves (38.9%). Compared to human resistomes, dissimilarity was highest for broiler and turkey (86.7 and 83.8%, respectively) and lowest for veal calves and pig (61.6 and 61.8%, respectively). The mean of all pairwise resistome dissimilarities was 66.9%. The top 10 AMR determinant contributors to each reservoir pairwise dissimilarity are indicated in Supplementary Table 4. These ten AMR determinants always contributed cumulatively above 50% (mean = 62%, standard deviation = 6%) to overall dissimilarity between two reservoirs, and we did not observe a relationship between the average pairwise reservoir dissimilarity percentage and the total number of determinants contributing to it (Supplementary Figure 3).

**FIGURE 5:**
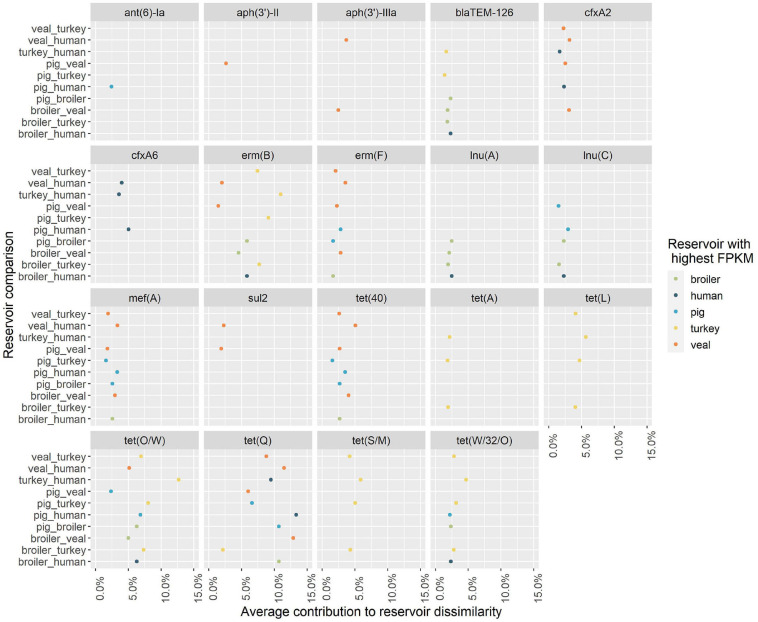
Antimicrobial resistance determinants with top contribution to pairwise dissimilarity between the resistomes of five reservoirs. The contribution of individual AMR determinants to the dissimilarity between every two reservoirs. The genes indicated are the representative genes for the AMR determinants identified among the top contributors for reservoir dissimilarity (see Supplementary Table 1 for further information on the composition of each AMR determinant). The color of the points represents the reservoir for which the AMR determinant had a higher relative abundance (Fragments Per Kilobase of reference and Million reads (FPKM)) in a pairwise comparison. The horizontal axis represents the average proportional contribution of an AMR determinant to the overall average dissimilarity between two reservoirs. The vertical axis shows the two reservoirs compared.

**FIGURE 6:**
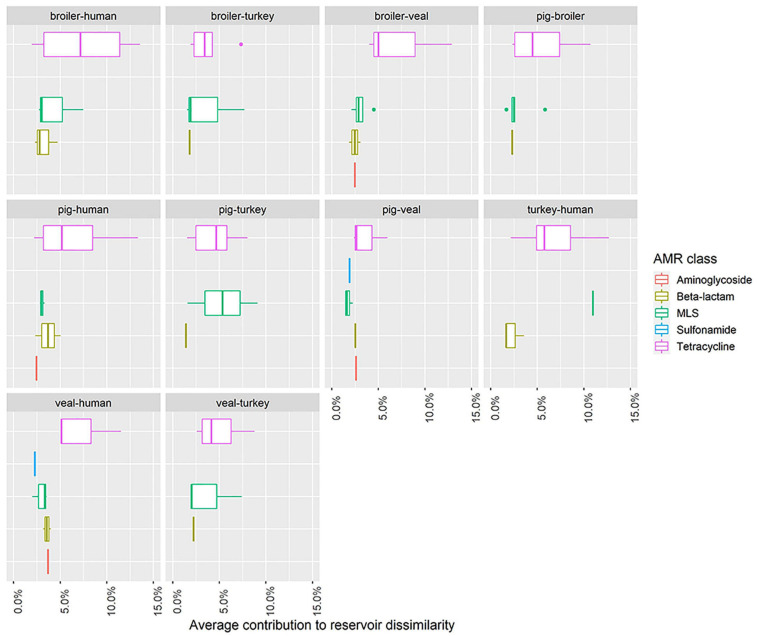
Distribution within antimicrobial class of the average contribution of individual AMR determinants to pairwise dissimilarity between the resistomes of five reservoirs. Each boxplot represents the distribution of the contribution to dissimilarity among AMR determinants (top contributors) of the same antimicrobial class. The five illustrated antimicrobial classes represent those that overall mostly contributed for the dissimilarity between resistomes of different reservoirs. Each plot facet represents a comparison between two reservoirs. The horizontal axis shows values of the average contribution of AMR determinants to pairwise dissimilarity.

Nineteen unique AMR determinants were identified among the top contributors in all one-to-one reservoir comparisons (Figure 5). For each occurrence of one of those unique determinants in a pairwise comparison, we investigated in which reservoir its mean abundance was highest. Figure 5 shows in which comparisons determinants have been seen as top contributors, and for which reservoirs they have been identified with highest relative abundance. Some of the top contributors have been identified as resistome signatures exclusive of a single reservoir. These included the genes (and their 90% homologous) *aph(3’)-II*, *aph(3’)-IIIa* and *sul2* as markers of veal calves resistomes, *tet(A)*, *tet(L)* and *tet(S/M)* as markers of turkey resistomes, *ant(6)-Ia* as marker of pig resistomes, *lnu(A)* as marker of broiler resistomes and *cfxA6* as marker of human resistomes. Additionally, *erm(F)*, *mef(A)*, *tet(40)* and *tet(Q)* were identified as resistome signatures primarily in veal calves, and secondly in pigs in comparison with other reservoirs, while *blaTEM-126* and *erm(B)* were signatures primarily in broilers or turkeys. The determinant *cfxA2* was identified as a marker of veal calves resistomes primarily and secondarily of human resistomes. Figure 6 shows the contribution of each AMR class to the average dissimilarity between every two reservoirs, considering the 19 top contributing AMR determinants. The classes that mostly contributed for the dissimilarity between resistomes of different reservoirs were, by descending order, tetracycline, macrolide, beta-lactam, aminoglycoside and sulfonamide. These classes were all represented among the AMR determinants with top contribution to pairwise dissimilarities.

**FIGURE 7:**
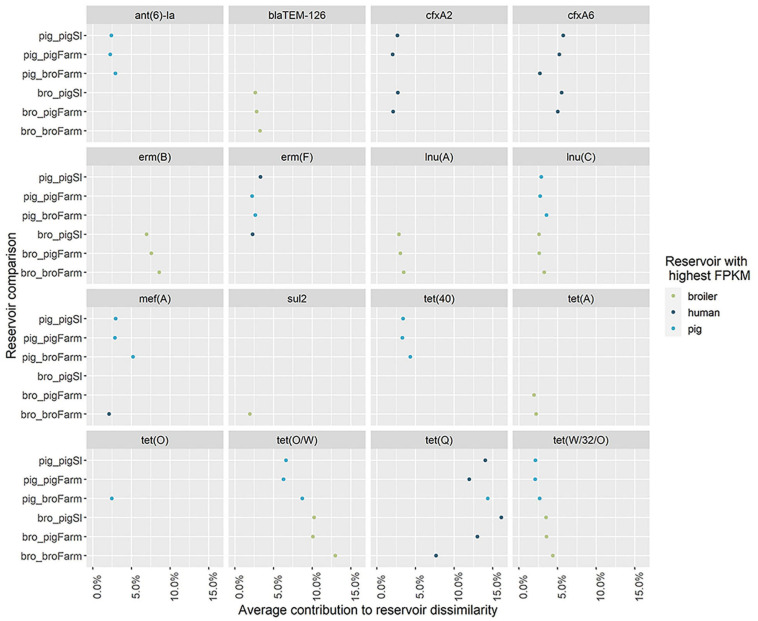
Antimicrobial resistance determinants with top contribution to pairwise dissimilarity between human resistomes and the resistomes of pigs and broilers. The contribution of individual AMR determinants to the dissimilarity between the resistomes of humans and those of pigs and broilers. The genes indicated are the representative genes for the AMR determinants identified among the top contributors for reservoir dissimilarity, in the similarity percentage analysis between pigs/broilers and three groups of sampled humans – workers on pig farms, pig slaughterhouses and broiler farms (see Supplementary Table 1 for further information on the composition of each AMR determinant). The color of the points represents the reservoir for which the AMR determinant had a higher relative abundance (Fragments Per Kilobase of reference and Million reads (FPKM)) in a pairwise comparison. The horizontal axis represents the average proportional contribution of an AMR determinant to the overall average dissimilarity between the resistomes of two reservoirs. The vertical axis represents the two reservoirs compared (‘pigSl’ = pig slaughterhouse workers; ‘pigFarm’ = pig farm workers; ‘broFarm’ = broiler farm workers).

**FIGURE 8:**
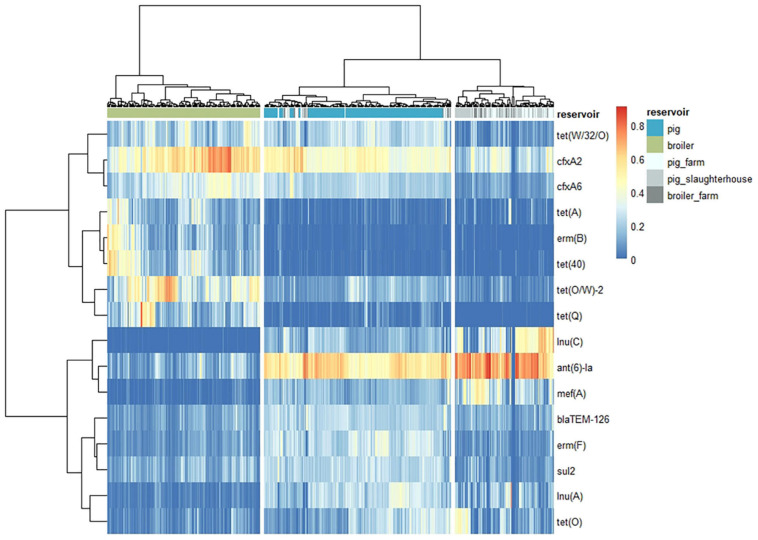
Distance between the resistomes of three groups of humans and the resistomes of pigs and broilers, based on the relative abundance of AMR features top contributors for reservoir pairwise dissimilarity. The heatmap represents vertical clustering of resistomes (Ward.D2 agglomeration method) based on the euclidean distance of the hellinger-transformed values of Fragments Per Kilobase of reference and Million reads (FPKM) of the AMR determinants indicated (horizontally clustered by correlation). These AMR determinants represent the 16 top contributors for the pairwise dissimilarities between the reservoirs “pig” and “broiler” and each of the three groups of humans occupationally exposed to direct contact with animals (workers on “pig_farm,” “pig_slaughterhouse,” or “broiler_farm”).

The dissimilarity analysis performed separately for each human group against pigs and broilers (Supplementary Table 5) showed that the overall dissimilarity between pig resistomes and the resistomes of pig farm workers was 8 to 12% lower (56%) when compared to the dissimilarity to the other two human groups (64% and 68.0%). In opposition, there was negligible difference (2 to 3%) in the dissimilarity between broiler resistomes and the resistomes of broiler farm workers (87%) compared to the dissimilarity between broilers and humans with direct contact to pigs (84% and 89%). Further differences between resistomes of subgroups within each group of workers here considered, i.e., farmers, their family members and employees, were previously described elsewhere (Van Gompel et al., 2020). Sixteen unique AMR determinants were identified among the top 10 contributors in all pairwise comparisons with the three human groups (Figure 7). Each of those determinants was identified as a ‘pig,’ ‘broiler,’ or ‘human’ resistome signature, when identified as consistently more abundant in those reservoirs. The determinants *ant(6)-Ia*, *mef(A)* and *tet(40)* were identified as ‘pig’ signatures, *blaTEM-126*, *lnu(A)* and *erm(B)* as ‘broiler’ signatures and *cfxA2*, *cfxA6* and *tet(Q)* as ‘human’ resistome signatures. Three determinants – *lnu(C)*, *tet(O/W)* and *tet(W/32/O)* – were always more abundant in the animal resistomes than in the human resistomes. Figure 8 shows the hierarchical clustering of samples based on the relative abundance of those 16 determinants. Overall, there was a clear separation between human, pig and broiler resistomes in three separate clusters. Most humans from the three occupational exposure settings cluster together in one cluster, however, with more proximity to pig resistomes. Some human samples overlap with the ‘pig cluster’ and no human sample clusters together with broiler resistomes. Most human samples overlapping with the ‘pig cluster’ belong to the group of pig farm workers.

In order to assess the significance of the contribution of individual AMR determinants to the pairwise dissimilarities here investigated, multiple regression studies are needed including the abundance of selected determinants as predictors of reservoir for each pairwise combination. Additionally, the significance of the reservoir signatures here identified can be further determined via discriminant analysis.

## Discussion

Supervised classification algorithms have been increasingly applied to trace back particular microbial contamination (Knights et al., 2011b; Henry et al., 2016; McCarthy et al., 2017) or antimicrobial resistance genes (Baral et al., 2018; Li et al., 2018; Gupta et al., 2019) in complex samples to different upstream sources in the context of environmental contamination. Random Forests has often been considered a reference algorithm against which other methods are benchmarked, usually being among the top performers (Knights et al., 2011a, b; Gupta et al., 2019). In this study, we explored the use of both flat and hierarchical Random Forests to attribute the source of AMR determinants to different reservoirs.

One of the main challenges in the application of classification algorithms to highly dimensional data, such as metagenomics, is the identification of the set of features that results in the ideal discrimination level for future predictions (Knights et al., 2011a). This challenge is exacerbated when features are not endemic to a single class (or source) (Knights et al., 2011b). Random Forests provides a ranking of each feature’s importance, which can be critically used to inform feature selection. In this study, the first step of feature selection was based on the importance of each AMR determinant in a first hierarchical Random Forests that accounted for separation of samples according to country of origin during model training. Other algorithms may have the advantage of embedding a feature selection step and therefore can be tuned to identify the minimum number of features that guarantees a defined level of overall accuracy (Knights et al., 2011a). It has been shown that the feature selection method itself can influence the model’s classification accuracy (Gupta et al., 2019). Future studies are thus needed to assess the performance of alternative algorithms and feature selection approaches.

Applying a hierarchical model, with ‘country’ as one of the hierarchy levels, allowed, however, not only to reduce the number of features in subsequent steps, but also to identify country-specific and country-independent AMR determinants with a positive impact on the classification of animal resistomes into corresponding reservoirs. This step was important to subsequently define and assess a general source-attribution model, independent of the country of origin of the reservoirs. When assessing the performance of the first hierarchical Random Forests model, we observed that the number of AMR determinants necessary to classify a resistome across countries, i.e., in a country-independent manner, increases with a larger heterogeneity of reservoirs among the samples. While 35 AMR determinants could be coincidently used to attribute a resistome to the sources ‘pig’ or ‘broiler’ across five countries, our method selected 119 determinants to differentiate between the resistomes of pigs, broilers, turkeys and veal calves across two countries only. The reduction of predictor features to those most country-independent in a second hierarchical Random Forests model did not result in an overall improvement in model performance. However, improvements in classification were observed individually for resistomes originating in countries A, E and I. For these countries, there was a decrease in OOB error and/or an increase in percentage concordance between predicted- and true- country class and improved accuracy in the prediction of two reservoir classes. Contrarily, classification accuracy overall decreased for countries G, D and F, suggesting that the impact of country-specific resistome signatures in prediction may be higher in these countries. The performance of the second hierarchical Random Forests also showed an improvement in the classification of resistomes of broilers and turkeys, opposite to a decrease in prediction accuracy for resistomes of veal calves and pigs.

We built three flat Random Forests models under different hypotheses and assessed their differences in prediction performance. The first model, trained exclusively on animal resistomes, proved obviously inadequate to correctly attribute the source of human resistomes, illustrating the need to account for an ‘unknown,’ ‘other,’ or ‘human’ source while building a source attribution of antimicrobial resistance. However, the predictions of this model were in accordance with those of the second Random Forests, in the fact that they both indicate that among the four livestock species considered, pigs seem to have the resistome composition closest to the composition of the human resistome. The third model, trained on observations of a single country and on a set of country-specific resistome signatures, showed a slight increase in accuracy during training compared to the previous (country-independent model) and a decrease in the proportion of votes attributed to ‘pig’ among samples collected from broiler farm workers. This finding supports the indication that occupational exposure to AMR determinants was higher among workers exposed to pigs than workers of broiler farms. Despite that difference between the models, both showed that workers on pig farms had resistomes more often attributed to the source ‘pig’ than workers in pig slaughterhouses. Furthermore, the country-specific model showed that, selecting the appropriate resistome signatures of the reservoirs sampled in that country, may be needed in order to improve attribution results and detect or strengthen the evidence for particular transmission links. This may not, however, be necessarily true for every country. For example, an overall improvement in prediction was observed for countries A, E and I when the feature space was reduced to a set of country-independent resistome signatures. The fact that country F was more difficult to classify with the same feature space may indicate that the country-independent set of features did not include important resistome signatures for source differentiation within this country.

The results of the dissimilarity analysis between reservoirs clearly showed that human resistomes have a composition closer to resistomes of pigs and veal calves than to those of broilers and turkeys. Surprisingly, resistomes of the two poultry species were less similar than the resistomes of pigs and veal calves. Further studies that investigate differences in the microbiome of and account for antimicrobial use practices in different reservoirs may help to elucidate the dissimilarities here estimated. However, two recent studies have shown differences in the susceptibility to antibiotics of *Lactobacillus* isolated from chickens (Dec et al., 2017) and from turkeys (Dec et al., 2018) in the same country. While isolates from both species presented high resistance prevalence against tetracycline, lincomycin and enrofloxacin, isolates from chicken presented a higher prevalence of resistance to tiamulin. Chicken-derived isolates were also more often multidrug resistant than turkey-derived isolates, and while the resistance gene *erm(B)* was highly prevalent in both cases, the gene *lnu(A)* was more commonly found among chicken-derived isolates.

At least 50% of the resistome dissimilarity between every two reservoirs included in our study can be explained by a set of 10 AMR determinants. Among these top contributors to dissimilarity, we identified resistome signatures for each individual reservoir. These signatures are to be considered generally country-independent, since the analysis was based on the full set of 389 AMR determinants. We also assessed the similarity of the resistome of each human group (workers on pig farms, pig slaughterhouses or broiler farms) to the resistomes of broilers and pigs. The results clearly showed that the overall dissimilarity between pig resistomes and the resistomes of pig farm workers was lower when compared to the dissimilarity to the other two human groups, while dissimilarity between broiler resistomes and the resistomes of the three groups was similar. This finding suggests the occurrence of transmission of AMR determinants between workers and pigs within the farm environment. This is also supported by the clustering of samples based on the relative abundance of AMR determinants with a top contribution to dissimilarity, since samples from pig farm workers are those occasionally overlapping with samples from pigs. Resistome signatures found for pigs and broilers in this study included resistance genes previously also identified as elements of the ‘core resistome’ of those species, using the same set of samples (Munk et al., 2018). AMR determinants identified here as exclusive (*lnu(A)*) or predominant (*blaTEM-126*) markers for the reservoir broiler were also identified as exclusive of this reservoir’s core resistome in that study. In another study, the determinant *lnu(A)* was also one of the most prevalent (39%) AMR genes identified in *Lactobacillus* isolated from chicken (Dec et al., 2017). Genes here identified as exclusive (*ant(6)-Ia*) or predominant (*erm(F)*, *mef(A)*, *tet(40)*, *tet(Q)*) markers of pig resistomes, were previously identified as part of both broiler’s and pig’s core resistomes. This difference between studies in the exclusive association of AMR determinants to a single reservoir’s resistome could be due to the fact that our study additionally included comparison to the resistomes of turkeys and veal calves.

The same pig and broiler resistomes have also been recently assessed for associations with lifetime antimicrobial use at farm level. Van Gompel et al. (2019) found significant positive associations between the use of lincosamide/macrolide, tetracycline or macrolide antimicrobials on pig farms and the relative abundance of the clusters *erm(F)*, *tet(40)*, or *mef(A)*, respectively. Here, we identified these three AMR determinants as resistome markers predominantly in pigs. Luiken et al. (2019) found positive significant associations between the group treatments macrolide/lincosamide/streptogramin or beta-lactam antimicrobials on broiler farms, and the relative abundance of *erm(B)* or *blaTEM* clusters, respectively. We identified both AMR determinants as resistome markers predominantly in broilers. The interpretation of our findings in the context of those association studies suggests that the continued and generalized use of certain antimicrobials in specific production settings eventually will lead to the definition of the resistome composition, not only within individual farms or individual countries, but ultimately at a reservoir level, and may ultimately influence the dynamics of the transmission of antimicrobial resistance from livestock animals to humans.

The two antimicrobial determinants, *cfxA2* and *cfxA6*, that we identified here as resistome markers of the humans included in the study, have been identified in β-lactamase-producing oral anaerobic bacteria such as *Prevotella* spp. and *Bacteroides* spp. (Binta and Patel, 2016).

In accordance with our findings, a study analyzing the same human occupational populations (Van Gompel et al., 2020) previously demonstrated higher abundance of AMR genes in pig exposed workers compared to broiler exposed workers and control subjects from the same country (persons without occupational contact). That study also identified significant between- and within occupational group resistome compositional differences. The number of on-farm working hours and working on pig a farm (compared to working on a broiler farm) were found to be associated with the presence of specific abundances of AMR genes in human stools, also suggesting potential livestock-associated resistome acquisition in humans. In addition, resistome of pig exposed workers were compared in a differential abundance analysis with those of the control subjects. The latter identified significantly different fecal abundances of 30 AMR genes, including the increased abundance of *mef(A)*, *tet(Q)*, *erm(F)* and *tet(40)*, which were here identified as predominantly originating from pigs.

In this study, we showed that it is possible to use metagenomics data to attribute the occurrence of fecal antimicrobial resistance in humans to different livestock reservoirs. We used Random Forests algorithms and dissimilarity analysis (SIMPER) to identify country-specific and reservoir-specific resistome markers that can be used to design targeted source-attribution studies. The need for country-specific models needs to be assessed on a case-by-case basis, as countries may present different heterogeneity between reservoirs’ resistomes. In one of the countries, the results suggested a link between occupational exposure to pigs and the presence in human stools of antimicrobial resistance determinants mainly associated with pigs. This finding must be interpreted with caution, since gut resistome is expected to be influenced to some degree by gut microbiome composition, and among the reservoirs included in the study, pigs may have the gut microbiota closest to humans. Further studies are needed considering the relationship between microbiome and resistome in the different species and assessing the microbiome similarity between humans and livestock reservoirs.

Additionally, some of the limitations of the present study need to be further addressed, including expanding the sampling of human resistomes to more than a single country and to the general human population (non-occupationally exposed to livestock), and collecting resistome samples of all livestock species considered for each country in the analysis. Furthermore, the number of samples collected for each population (e.g., reservoir) and subgroup within a population (e.g., different subgroups of humans) should be balanced.

Despite the need for future improvements, this study sets the scene for the implementation of metagenomics in source-attribution in the food chain, and thus for an alternative, resistome-based approach to assess transmission of antimicrobial resistance.

## Members of the Effort Consortium

Members of the EFFORT consortium: H. Graveland (2), D.J.J. Heederik (2), L.A.M. Smit (2), H. Schmitt (2), R.E.C. Luiken (2), D.J. Mevius (2,4), A. van Essen (4), D. Ceccarelli (4), A. Hesp (4), J. van der Goot (4), B. Gonzalez- Zorn (6), G. Moyano (6), P. Sanders (7), C. Chauvin (7), J. David (7), A. Battisti (8), A. Caprioli (8), T. Blaha (9), K. Wadepohl (9), M. Brandt (9), P. Munk (1), D. Wasyl (10), M. Skarżyńska (10), M. Zajaç (10), H. Daskalov (11), H.W. Saatkamp (12), K.D.C. Stärk (13), P. Joosten (14), S. Sarrazin (14), J. Dewulf (14).

(6) Complutense University of Madrid, Madrid, Spain; (7) French Agency for Food, Environmental and Occupational Health and Safety, Fougères, France; (8) Istituto Zooprofilattico Sperimentale del Lazio e della Toscana, Rome, Italy; (9) University of Veterinary Medicine Hannover, Bakum, Germany; (10) National Veterinary Research Institute, Pulawy, Poland; (11) National Diagnostic Research Veterinary Institute, Sofia, Bulgaria; (12) Wageningen University and Research, Wageningen, the Netherlands; (13) SAFOSO AG, Liebefeld, Switzerland; (14) Ghent University, Merelbeke, Belgium.

## Data Availability Statement

The datasets presented in this study can be found in online repositories. The names of the repository/repositories and accession number(s) can be found below: https://www.ebi.ac.uk/ena, PRJEB22062, https://www.ebi.ac.uk/ena, PRJEB39685, and https://ega-archive.org/studies, ID EGAS00001003944.

## Ethics Statement

The studies involving human participants were reviewed and approved by the Medical Ethical Committee of the University Medical Centre Utrecht (NL) which confirmed that the Dutch ‘Medical Research Involving Human Subjects Act’ did not apply for the study of the EFFORT human populations (Protocols14–346/C, 14–403/C) The patients/participants provided their written informed consent to participate in this study.

## Author Contributions

AD: conceptualization, methodology, formal analysis, investigation, visualization, project administration, and writing of original draft. TR, LV, and AB: resources, data curation, and writing – review and editing. TP, RH, and IH: resources and writing – review and editing. FA: supervision and writing – review and editing. JW: funding acquisition, project administration, and writing – review and editing. TH: conceptualization, supervision, project administration, and writing – review and editing. All authors contributed to the article and approved the submitted version.

## Conflict of Interest

The authors declare that the research was conducted in the absence of any commercial or financial relationships that could be construed as a potential conflict of interest.

## Footnotes

1 EFFORT – Ecology from Farm to Fork Of microbial drug Resistance and Transmission (http://www.effort-against-amr.eu/).

2 Picard – https://broadinstitute.github.io/picard

3 BBduk2 – https://sourceforge.net/projects/bbmap

4 ResFinder database – https://bitbucket.org/genomicepidemiology/resfinder (accessed September 21, 2018).

5 HieRanFor package development page – https://r-forge.r-project.org/R/?group_id=2021

6 https://ec.europa.eu/eurostat/data/database

7 https://comtrade.un.org/
