# Supplementary material for: Metagenomics-Based Approach to Source-Attribution of Antimicrobial Resistance Determinants – Identification of Reservoir Resistome Signatures
Source: Front Microbiol. 2021 Jan 15;11:601407. doi: 10.3389/fmicb.2020.601407 (PMC7843941; doi:10.3389/fmicb.2020.601407)
Supplement: Supplementary file 3 [file Data_Sheet_1.PDF]

Supplementary Material

1 Supplementary Data

Supplementary Data 1. The DNA sequences (reads) from the metagenomic samples collected from animals are deposited in the European Nucleotide Archive under the project accession numbers PRJEB22062 (pig and broiler samples), PRJEB39685 (veal calves and turkey samples) and those from the metagenomic samples collected from humans are deposited in the European Genome-Phenome Archive with the study ID EGAS00001003944 and dataset ID EGAD00001005444.

2 Supplementary Figures and Tables

2.1 Supplementary Tables

Supplementary Table 1. Composition of AMR determinants (AMR genes clustered by 90% homology).

For each AMR determinant (‘cluster ID’), the corresponding representative resistance gene, and the list of individual genes contained in the cluster.

Supplementary Table 1 is provided in a separate Excel file.

Model	Description	HierarchyLevels	FeatureSpace (P)	mtry (sqrt(P))	ReservoirsTraining	Countries	Accuracy*†	Kappa†
HRF1	Hierarchical Random Forest	Country, Reservoir	full (389)	20	pig, broiler, veal calves, turkey	all	0.833	0.825
HRF2	Hierarchical Random Forest	Country, Reservoir	reduced (119)	11	pig, broiler, veal calves, turkey	all	0.826	0.844
RF1	Random Forest	Reservoir	reduced (119)	11	pig, broiler, veal calves, turkey	all	0.996	0.995
RF2	Random Forest	Reservoir	reduced (119)	11	human, pig, broiler, veal calves, turkey	all	0.995	0.994
RF3	Random Forest	Reservoir	reduced (109)	10	human, pig, broiler, veal calves, turkey	F[#]	1	1

*Accuracy – observed accuracy of classification during training; †mean of 100 model iterations; [#]model RF3 was specific for country F but included surrogate turkey samples from country B.

Supplementary Table 2. Overview of model characteristics and performance during training.

Supplementary Table 3. Composition of country-specific clusters and country-independent clusters (intersection between countries) of AMR determinants predictors of reservoir. The table lists the country-specific and country-independent AMR determinants, outputs of the Venn diagrams (Supplementary Figure 1), for the intersection between (A) two countries with sample collection in pig-, broiler-, veal calves- and turkey- farms; (B) four countries with sample collection in pig-, broiler-, veal calves- and/or turkey- farms; (C) five countries with sample collection in pig- and broiler- farms. For each AMR determinant, the representative resistance gene is also indicated.

Supplementary Table 3 (A), (B) and (C) are provided in a separate Excel file.

AMR determinant ID	AMR class	Representative AMR gene	Phenotypic resistance*	Average dissimilarity [a]	Standard deviation [b]	Cumulative contribution[c]	Mean FPKM A	Mean FPKM B
**Overall average dissimilarity between pig (A) and broiler (B): 72%**								
21	Tetracycline	*tet(Q)*	Doxycycline, Tetracycline, Minocycline	11%	5%	15%	**138.83**	8.68
29	Tetracycline	*tet(O/W)-2_1*	Doxycycline, Tetracycline, Minocycline	6%	6%	24%	85.68	**132.79**
454	Macrolide, Lincosamide, Streptogramin B	*erm(B)*	Erythromycin, Lincomycin, Clindamycin, Quinupristin, Pristinamycin IA, Virginiamycin S	6%	7%	32%	13.49	**88.93**
113	Tetracycline	*tet(40)*	Doxycycline, Tetracycline	3%	2%	35%	**37.12**	2.86
114	Macrolide	*mef(A)*	Erythromycin, Azithromycin	3%	2%	39%	**33.08**	1.05
611	Lincosamide	*lnu(A)*	Lincomycin	3%	4%	42%	1.85	**35.75**
32	Tetracycline	*tet(W/32/O)*	Doxycycline, Tetracycline, Minocycline	2%	2%	46%	24.88	**42.39**
93	Beta-lactam	*blaTEM-126*	Amoxicillin, Ampicillin, Aztreonam, Cefepime, Cefotaxime, Ceftazidime, Ceftriaxone, Piperacillin, Ticarcillin	2%	3%	49%	0.43	**33.42**
605	Llincosamide	*lnu(C)*	Lincomycin	2%	2%	52%	29.88	**32.01**
408	Macrolide, Lincosamide, Streptogramin B	*erm(F)*	Erythromycin, Lincomycin, Clindamycin, Quinupristin,	2%	2%	54%	**23.75**	2.70

			Pristinamycin IA, Virginiamycin S					
**Overall average dissimilarity between pig (A) and veal calves (B): 39%**								
21	Tetracycline	*tet(Q)*	Doxycycline, Tetracycline, Minocycline	6%	4%	15%	138.83	**193.85**
113	Tetracycline	*tet(40)*	Doxycycline, Tetracycline	3%	2%	22%	37.12	**59.79**
425	Aminoglycoside	*aph(3')-IIIa*	Amikacin	3%	1%	29%	11.67	**43.05**
189	Beta-lactam	*cfxA2*	Amoxicillin, Ampicillin, Cefotaxime, Ceftazidime, Ceftriaxone, Piperacillin, Ticarcillin	3%	1%	36%	11.28	**44.58**
29	Tetracycline	*tet(O/W)-2*	Doxycycline, Tetracycline, Minocycline	2%	2%	42%	**85.68**	82.13
408	Macrolide, Lincosamide, Streptogramin B	*erm(F)*	Erythromycin, Lincomycin, Clindamycin, Quinupristin, Pristinamycin IA, Virginiamycin S	2%	2%	48%	23.75	**43.34**
316	Sulfonamide	*sul2*	Sulfamethoxazole	2%	1%	52%	0.76	**26.06**
114	Macrolide	*mef(A)*	Erythromycin, Azithromycin	2%	1%	57%	33.08	**43.18**
605	Lincosamide	*lnu(C)*	Lincomycin	2%	1%	61%	**29.88**	9.54
454	Macrolide, Lincosamide, Streptogramin B	*erm(B)*	Erythromycin, Lincomycin, Clindamycin, Quinupristin, Pristinamycin IA, Virginiamycin S	2%	1%	65%	13.49	**25.08**
**Overall average dissimilarity between pig (A) and turkey (B): 67 %**								
454	Macrolide, Lincosamide, Streptogramin B	*erm(B)*	Erythromycin, Lincomycin, Clindamycin, Quinupristin, Pristinamycin IA, Virginiamycin S	9%	6%	14%	13.49	**178.37**

29	Tetracycline	*tet(O/W)-2*	Doxycycline, Tetracycline, Minocycline	8%	6%	26%	85.68	**222.19**
21	Tetracycline	*tet(Q)*	Doxycycline, Tetracycline, Minocycline	7%	4%	35%	**138.83**	36.69
25	Tetracycline	*tet(S/M)*	Doxycycline, Tetracycline, Minocycline	5%	3%	43%	2.33	**91.42**
90	Tetracycline	*tet(L)*	Doxycycline, Tetracycline	5%	3%	50%	7.06	**87.16**
32	Tetracycline	*tet(W/32/O)*	Doxycycline, Tetracycline, Minocycline	3%	2%	55%	24.88	**76.70**
96	Tetracycline	*tet(A)*	Doxycycline, Tetracycline	2%	2%	57%	0.24	**30.43**
114	Macrolide	*mef(A)*	Erythromycin, Azithromycin	2%	1%	60%	**33.08**	9.12
113	Tetracycline	*tet(40)*	Doxycycline, Tetracycline	2%	2%	62%	**37.12**	14.13
293	Beta-lactam	*blaTEM-126*	Amoxicillin, Ampicillin, Aztreonam, Cefepime, Cefotaxime, Ceftazidime, Ceftriaxone, Piperacillin, Ticarcillin	1%	2%	64%	0.43	**22.93**
**Overall average dissimilarity between broiler (A) and veal calves (B): 72%**								
21	Tetracycline	*tet(Q)*	Doxycycline, Tetracycline, Minocycline	13%	4%	18%	8.68	**193.85**
29	Tetracycline	*tet(O/W)-2*	Doxycycline, Tetracycline, Minocycline	5%	4%	25%	**132.79**	82.13
454	Macrolide, Lincosamide, Streptogramin B	*erm(B)*	Erythromycin, Lincomycin, Clindamycin, Quinupristin, Pristinamycin IA, Virginiamycin S	5%	6%	31%	**88.93**	25.08

113	Tetracycline	*tet(40)*	Doxycycline, Tetracycline	4%	2%	37%	2.86	**59.79**
189	Beta-lactam	*cfxA2*	Amoxicillin, Ampicillin, Cefotaxime, Ceftazidime, Ceftriaxone, Piperacillin, Ticarcillin	3%	1%	41%	0.46	**44.58**
114	Macrolide	*mef(A)*	Erythromycin, Azithromycin	3%	1%	45%	1.05	**43.18**
408	Macrolide, Lincosamide, Streptogramin B	*erm(F)*	Erythromycin, Lincomycin, Clindamycin, Quinupristin, Pristinamycin IA, Virginiamycin S	3%	1%	49%	2.70	**43.34**
425	Aminoglycoside	*aph(3')-IIIa*	Amikacin	3%	1%	52%	7.89	**43.05**
611	Llincosamide	*lnu(A)*	Lincomycin	2%	4%	55%	**35.75**	0.85
293	Beta-lactam	*blaTEM-126*	Amoxicillin, Ampicillin, Aztreonam, Cefepime, Cefotaxime, Ceftazidime, Ceftriaxone, Piperacillin, Ticarcillin	2%	2%	58%	**33.42**	1.75
**Overall average dissimilarity between broiler (A) and turkey (B): 59%**								
454	Macrolide, Lincosamide, Streptogramin B	*erm(B)*	Erythromycin, Lincomycin, Clindamycin, Quinupristin, Pristinamycin IA, Virginiamycin S	8%	6%	13%	88.93	**178.37**
29	Tetracycline	*tet(O/W)-2*	Doxycycline, Tetracycline, Minocycline	7%	6%	25%	132.79	**222.19**
25	Tetracycline	*tet(S/M)*	Doxycycline, Tetracycline, Minocycline	4%	3%	33%	17.63	**91.42**
90	Tetracycline	*tet(L)*	Doxycycline, Tetracycline	4%	3%	40%	18.33	**87.16**
32	Tetracycline	*tet(W/32/O)*	Doxycycline, Tetracycline, Minocycline	3%	2%	44%	42.39	**76.70**

21	Tetracycline	*tet(Q)*	Doxycycline, Tetracycline, Minocycline	2%	3%	48%	8.68	**36.69**
611	Lincosamide	*lnu(A)*	Lincomycin	2%	3%	51%	**35.75**	13.66
96	Tetracycline	*tet(A)*	Doxycycline, Tetracycline	2%	2%	54%	24.74	**30.43**
293	Beta-lactam	*blaTEM-126*	Amoxicillin, Ampicillin, Aztreonam, Cefepime, Cefotaxime, Ceftazidime, Ceftriaxone, Piperacillin, Ticarcillin	2%	2%	58%	**33.42**	22.93
605	Lincosamide	*lnu(C)*	Lincomycin	2%	2%	60%	**32.01**	15.92
**Overall average dissimilarity between veal calves (A) and turkey (B): 65%**								
21	Tetracycline	*tet(Q)*	Doxycycline, Tetracycline, Minocycline	9%	4%	14%	**193.85**	36.69
454	Macrolide, Lincosamide, Streptogramin B	*erm(B)*	Erythromycin, Lincomycin, Clindamycin, Quinupristin, Pristinamycin IA, Virginiamycin S	7%	5%	25%	25.08	**178.37**
29	Tetracycline	*tet(O/W)-2*	Doxycycline, Tetracycline, Minocycline	7%	5%	36%	82.13	**222.19**
25	Tetracycline	*tet(S/M)*	Doxycycline, Tetracycline, Minocycline	4%	3%	42%	6.83	**91.42**
90	Tetracycline	*tet(L)*	Doxycycline, Tetracycline	4%	3%	48%	5.52	**87.16**
32	Tetracycline	*tet(W/32/O)*	Doxycycline, Tetracycline, Minocycline	3%	2%	53%	18.47	**76.70**
113	Tetracycline	*tet(40)*	Doxycycline, Tetracycline	3%	1%	57%	**59.79**	14.13
189	Beta-lactam	*cfxA2*	Amoxicillin, Ampicillin, Cefotaxime, Ceftazidime, Ceftriaxone,	2%	1%	60%	**44.58**	4.56

			Piperacillin, Ticarcillin					
408	Macrolide, Lincosamide, Streptogramin B	*erm(F)*	Erythromycin, Lincomycin, Clindamycin, Quinupristin, Pristinamycin IA, Virginiamycin S	2%	1%	63%	**43.34**	6.65
114	Macrolide	*mef(A)*	Erythromycin, Azithromycin	2%	1%	66%	**43.18**	9.12

Supplementary table 4. AMR determinants with top contribution to pairwise dissimilarity between reservoirs. Contribution of individual AMR determinants (top 10) to the average overall Bray-Curtis dissimilarity between every two reservoirs. [a]AMR determinant contribution to average between-reservoir dissimilarity; [b] standard deviation of a; [c] ordered cumulative contribution of AMR determinant to average between-reservoir dissimilarity; [*]predicted phenotypic resistance according to the ResFinder database (Zankari et al. 2012), i.e. antibiotics to which the representative gene confers resistance, among a selected panel of (clinically relevant) antibiotics. FPKM represents relative abundance of AMR determinants measured in Fragments Per Kilobase of reference and Million reads mapped.

AMR determinant ID	AMR class	Representative AMR gene	Phenotypic resistance*	Average dissimilarity [a]	Standard deviation [b]	Cumulative contribution[c]	Mean FPKM A	Mean FPKM B
**Overall average dissimilarity between pig (A) and pig farm workers (B): 56%**								
21	Tetracycline	*tet(Q)*	Doxycycline, Tetracycline, Minocycline	12%	9%	21%	138.83	150.99
29	Tetracycline	*tet(O/W)-2*	Doxycycline, Tetracycline, Minocycline	6%	5%	32%	85.68	47.09
177	Beta-lactam	*cfxA6*	NA	5%	5%	42%	11.18	55.69
113	Tetracycline	*tet(40)*	Doxycycline, Tetracycline	3%	3%	48%	37.12	7.77
114	Macrolide	*mef(A)*	Erythromycin, Azithromycin	3%	2%	53%	33.08	12.95
605	Lincosamide	*lnu(C)*	Lincomycin	3%	2%	58%	29.88	6.02
282	Aminoglycoside	*ant(6)-Ia*	Streptomycin	2%	1%	62%	26.21	6.07
408	Macrolide, Lincosamide, Streptogramin B	*erm(F)*	Erythromycin, Lincomycin, Clindamycin, Quinupristin, Pristinamycin IA, Virginiamycin S	2%	2%	65%	23.75	6.65
32	Tetracycline	*tet(W/32/O)*	Doxycycline, Tetracycline, Minocycline	2%	2%	69%	24.88	6.79
189	Beta-lactam	*cfxA2*	Amoxicillin, Ampicillin, Cefotaxime, Ceftazidime, Ceftriaxone, Piperacillin, Ticarcillin	2%	2%	73%	11.28	22.65
**Overall average dissimilarity between pig (A) and pig slaughterhouse workers (B): 64%**								
21	Tetracycline	*tet(Q)*	Doxycycline, Tetracycline, Minocycline	14%	9%	22%	138.83	205.77
29	Tetracycline	*tet(O/W)-2*	Doxycycline, Tetracycline, Minocycline	7%	5%	32%	85.68	29.82

177	Beta-lactam	*cfxA6*	NA	6%	5%	41%	11.18	66.55
113	Tetracycline	*tet(40)*	Doxycycline, Tetracycline	3%	2%	47%	37.12	2.82
408	Macrolide, Lincosamide, Streptogramin B	*erm(F)*	Erythromycin, Lincomycin, Clindamycin, Quinupristin, Pristinamycin IA, Virginiamycin S	3%	3%	52%	23.75	30.95
114	Macrolide	*mef(A)*	Erythromycin, Azithromycin	3%	2%	57%	33.08	12.93
605	Lincosamide	*lnu(C)*	Lincomycin	3%	2%	61%	29.88	2.29
189	Beta-lactam	*cfxA2*	Amoxicillin, Ampicillin, Cefotaxime, Ceftazidime, Ceftriaxone, Piperacillin, Ticarcillin	3%	3%	65%	11.28	32.34
282	Aminoglycoside	*ant(6)-Ia*	Streptomycin	2%	1%	69%	26.21	3.27
32	Tetracycline	*tet(W/32/O)*	Doxycycline, Tetracycline, Minocycline	2%	2%	72%	24.88	4.10
**Overall average dissimilarity between pig (A) and broiler farm workers (B): 68%**								
21	Tetracycline	*tet(Q)*	Doxycycline, Tetracycline, Minocycline	14%	9%	21%	138.83	76.83
29	Tetracycline	*tet(O/W)-2*	Doxycycline, Tetracycline, Minocycline	9%	5%	34%	85.68	33.34
114	Macrolide	*mef(A)*	Erythromycin, Azithromycin	5%	7%	42%	33.08	27.31
113	Tetracycline	*tet(40)*	Doxycycline, Tetracycline	4%	3%	48%	37.12	2.48
605	Lincosamide	*lnu(C)*	Lincomycin	4%	2%	53%	29.88	7.12
282	Aminoglycoside	*ant(6)-Ia*	Streptomycin	3%	2%	57%	26.21	5.45
177	Beta-lactam	*cfxA6*	NA	3%	3%	61%	11.18	20.11

32	Tetracycline	*tet(W/32/O)*	Doxycycline, Tetracycline, Minocycline	3%	2%	65%	24.88	4.74
408	Macrolide, Lincosamide, Streptogramin B	*erm(F)*	Erythromycin, Lincomycin, Clindamycin, Quinupristin, Pristinamycin IA, Virginiamycin S	3%	2%	69%	23.75	6.04
28	Tetracycline	*tet(O)*	Doxycycline, Tetracycline, Minocycline	2%	1%	73%	22.39	5.50
**Overall average dissimilarity between broiler (A) and pig farm workers (B): 84%**								
21	Tetracycline	*tet(Q)*	Doxycycline, Tetracycline, Minocycline	13%	10%	15%	8.68	150.99
29	Tetracycline	*tet(O/W)-2*	Doxycycline, Tetracycline, Minocycline	10%	9%	28%	132.79	47.09
454	Macrolide, Lincosamide, Streptogramin B	*erm(B)*	Erythromycin, Lincomycin, Clindamycin, Quinupristin, Pristinamycin IA, Virginiamycin S	8%	8%	37%	88.93	1.48
177	Beta-lactam	*cfxA6*	NA	5%	5%	43%	0.03	55.69
32	Tetracycline	*tet(W/32/O)*	Doxycycline, Tetracycline, Minocycline	4%	3%	47%	42.39	6.79
611	Lincosamide	*lnu(A)*	Lincomycin	3%	5%	50%	35.75	0.02
293	Beta-lactam	*blaTEM-126*	Amoxicillin, Ampicillin, Aztreonam, Cefepime, Cefotaxime, Ceftazidime, Ceftriaxone, Piperacillin, Ticarcillin	3%	3%	54%	33.42	0.07
605	Lincosamide	*lnu(C)*	Lincomycin	3%	3%	57%	32.01	6.02
189	Beta-lactam	*cfxA2*	Amoxicillin, Ampicillin, Cefotaxime, Ceftazidime, Ceftriaxone,	2%	3%	59%	0.46	22.65

			Piperacillin, Ticarcillin					
96	Tetracycline	*tet(A)*	Doxycycline, Tetracycline	2%	3%	62%	24.74	0.06
**Overall average dissimilarity between broiler (A) and pig slaughterhouse workers (B): 89%**								
21	Tetracycline	*tet(Q)*	Doxycycline, Tetracycline, Minocycline	16%	11%	18%	8.68	205.77
29	Tetracycline	*tet(O/W)-2*	Doxycycline, Tetracycline, Minocycline	10%	9%	30%	132.79	29.82
454	Macrolide, Lincosamide, Streptogramin B	*erm(B)*	Erythromycin, Lincomycin, Clindamycin, Quinupristin, Pristinamycin IA, Virginiamycin S	7%	8%	37%	88.93	4.46
177	Beta-lactam	*cfxA6*	NA	6%	6%	44%	0.03	66.55
32	Tetracycline	*tet(W/32/O)*	Doxycycline, Tetracycline, Minocycline	4%	3%	48%	42.39	4.10
611	Lincosamide	*lnu(A)*	Lincomycin	3%	5%	51%	35.75	0.00
189	Beta-lactam	*cfxA2*	Amoxicillin, Ampicillin, Cefotaxime, Ceftazidime, Ceftriaxone, Piperacillin, Ticarcillin	3%	3%	54%	0.46	32.34
293	Beta-lactam	*blaTEM-126*	Amoxicillin, Ampicillin, Aztreonam, Cefepime, Cefotaxime, Ceftazidime, Ceftriaxone, Piperacillin, Ticarcillin	3%	3%	57%	33.42	0.32
605	Lincosamide	*lnu(C)*	Lincomycin	3%	3%	60%	32.01	2.29
408	Macrolide, Lincosamide, Streptogramin B	*erm(F)*	Erythromycin, Lincomycin, Clindamycin, Quinupristin, Pristinamycin IA, Virginiamycin S	2%	3%	62%	2.70	30.95

**Overall average dissimilarity between broiler (A) and broiler farm workers (B): 87%**								
29	Tetracycline	*tet(O/W)-2*	Doxycycline, Tetracycline, Minocycline	13%	11%	15%	132.79	33.34
454	Macrolide, Lincosamide, Streptogramin B	*erm(B)*	Erythromycin, Lincomycin, Clindamycin, Quinupristin, Pristinamycin IA, Virginiamycin S	9%	9%	25%	88.93	2.50
21	Tetracycline	*tet(Q)*	Doxycycline, Tetracycline, Minocycline	8%	9%	34%	8.68	76.83
32	Tetracycline	*tet(W/32/O)*	Doxycycline, Tetracycline, Minocycline	4%	4%	39%	42.39	4.74
611	Lincosamide	*lnu(A)*	Lincomycin	3%	6%	43%	35.75	0.03
605	Lincosamide	*lnu(C)*	Lincomycin	3%	4%	47%	32.01	7.12
293	Beta-lactam	*blaTEM-126*	Amoxicillin, Ampicillin, Aztreonam, Cefepime, Cefotaxime, Ceftazidime, Ceftriaxone, Piperacillin, Ticarcillin	3%	4%	50%	33.42	0.13
96	Tetracycline	*tet(A)*	Doxycycline, Tetracycline	2%	3%	53%	24.74	0.09
114	Macrolide	*mef(A)*	Erythromycin, Azithromycin	2%	7%	55%	1.05	27.31
316	Sulfonamide	*sul2*	Sulfamethoxazole	2%	2%	58%	18.72	0.73

Supplementary table 5. AMR determinants with top contribution to pairwise dissimilarity between pig/broiler resistomes and the resistomes of humans working in pigs farms/pig slaughterhouses/broiler farms. Contribution of individual AMR determinants (top 10) to the average overall Bray-Curtis dissimilarity between every two reservoir groups. [a]AMR determinant contribution to average between-reservoir group dissimilarity; [b] standard deviation of a; [c] ordered cumulative contribution of AMR determinant to average between-reservoir group dissimilarity; [*]predicted phenotypic resistance according to the ResFinder database (Zankari et al. 2012), i.e. antibiotics to which the representative gene confers resistance, among a selected panel of (clinically relevant) antibiotics. ‘FPKM’ represents relative abundance of AMR determinants measured in Fragments Per Kilobase of reference and Million reads mapped. ‘NA’ - The gene *cfxA6* is not annotated to a resistance phenotype in the database ResFinder.

2.2 Supplementary Figures

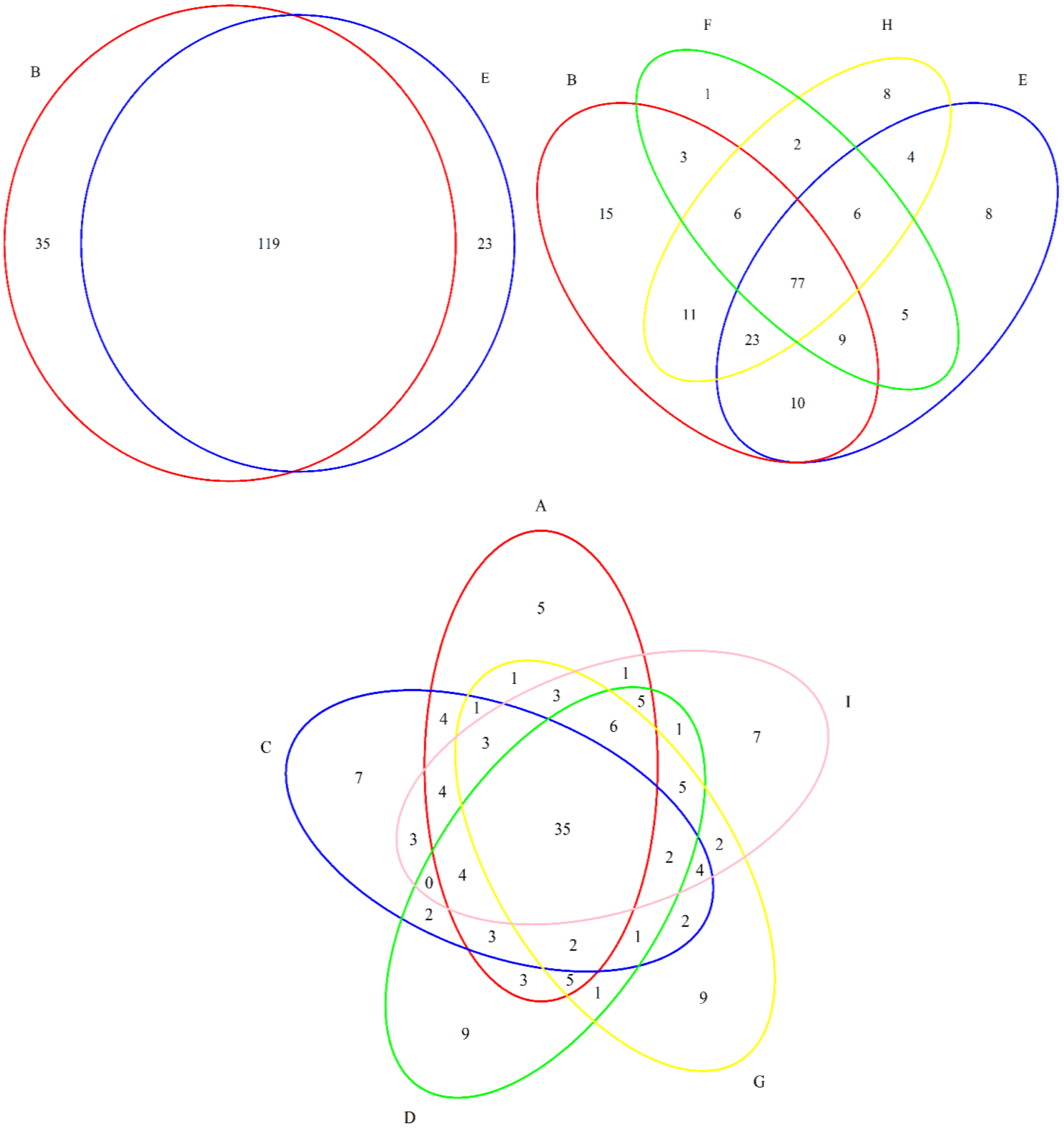

Supplementary Figure 1. Intersection between countries of AMR determinants predictors of reservoir. Venn diagrams showing the intersection between countries of AMR determinants with positive mean decrease in accuracy (MDA>0) in HRF1: intersection between two countries (B and E) including pig-, broiler-, turkey- and veal calves- resistomes; intersection between four countries (B, F, H and E) including pig-, broiler-, veal calves- or turkey- resistomes; intersection between five countries (C, A, I, G, D) including pig- and broiler- resistomes.

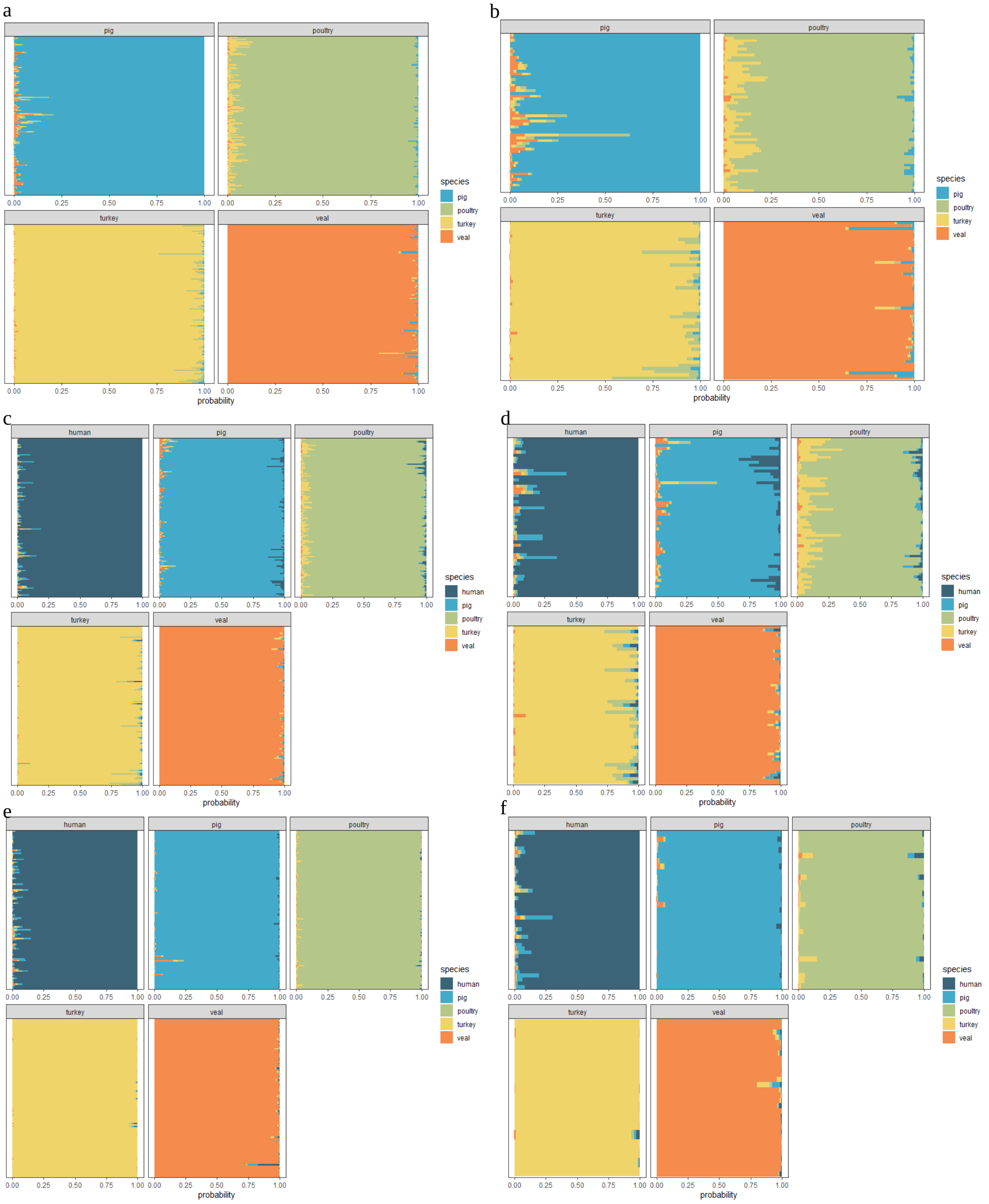

Supplementary Figure 2. Proportion of votes with test and training sets for RF1, RF2 and RF3. Relative attribution of resistomes to different reservoirs for training- (a,c,e) and testing- (b,d,f) datasets with models RF1(a,b), RF2(c,d) and RF3(e,f). Each facet represents the attribution predictions for resistomes of a given reservoir. The colors represent the attribution of a resistome to a given reservoir. The x-axis represents the probability of attribution.

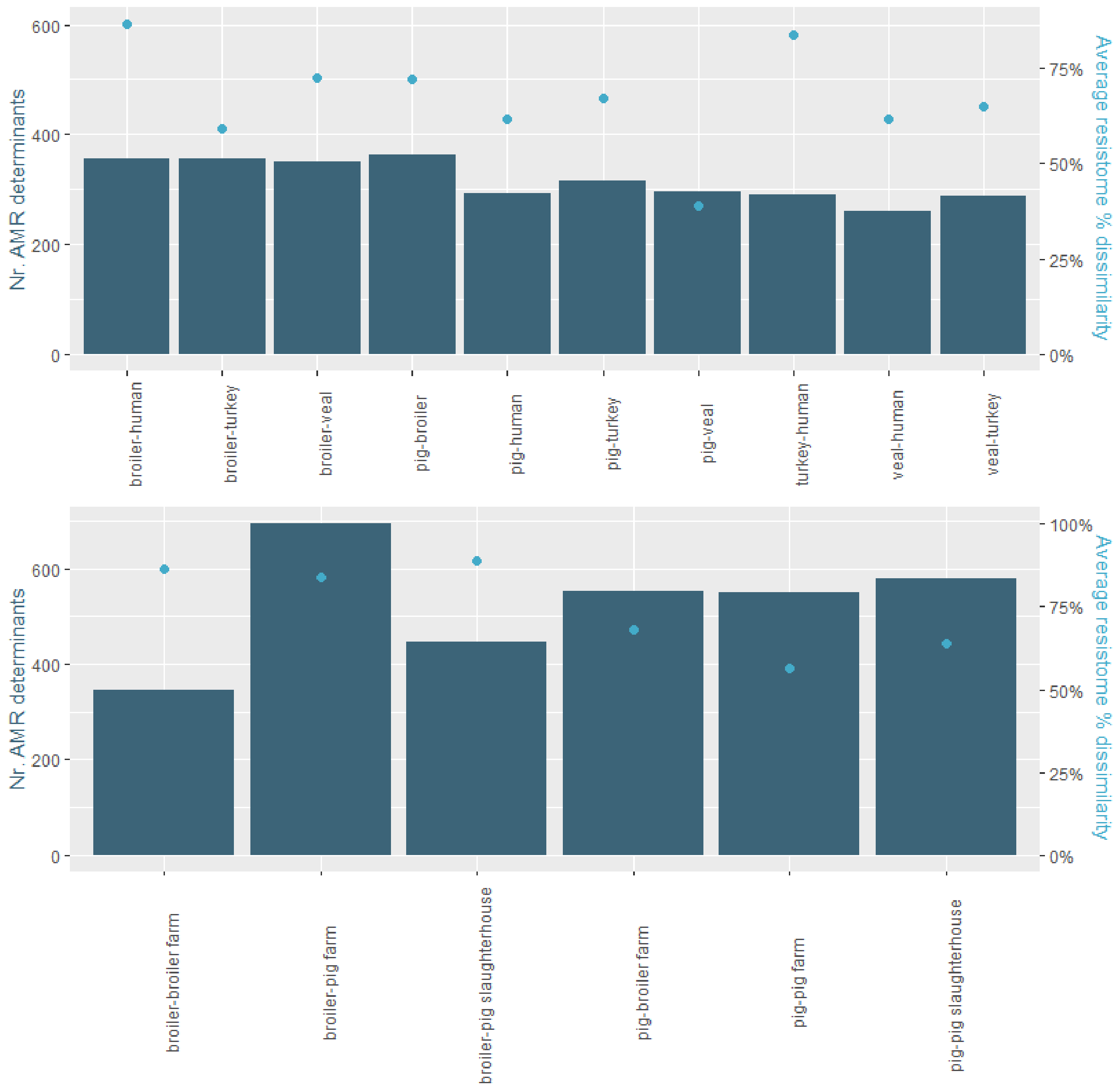

Supplementary Figure 3. Pairwise average resistome dissimilarity between reservoirs and total number of AMR determinants contributing to dissimilarity. The x-axis shows the pairwise-comparisons between reservoirs. The bars and primary y-axis show the number of AMR determinants contributing to average percent dissimilarity between two reservoirs. The dots and secondary y-axis represent the average percent dissimilarity between the resistomes of two reservoirs.

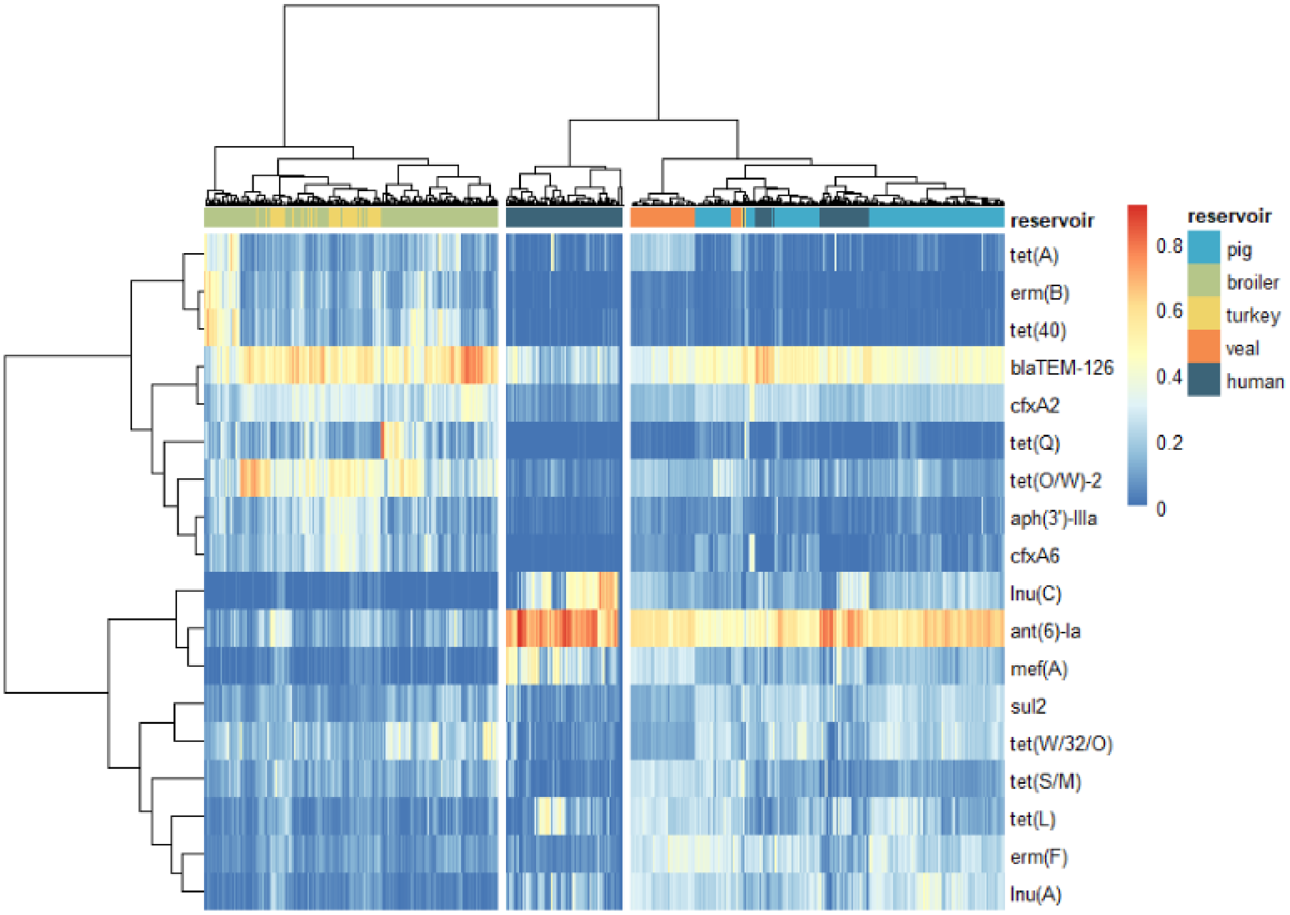

Supplementary Figure 4. Distance between the resistomes of five reservoirs based on the relative abundance of AMR determinants top contributors for reservoir pairwise dissimilarity. The heatmap represents vertical clustering of resistomes (Ward.D2 agglomeration method) based on the euclidean distance of the hellinger-transformed FPKM values. AMR determinants are horizontally clustered by correlation.
